# A portable system with sample rate of 250 Hz for characterization of knee and hip angles in the sagittal plane during gait

**DOI:** 10.1186/1475-925X-13-34

**Published:** 2014-03-31

**Authors:** Fermín Martínez-Solís, Abraham Claudio-Sánchez, José M Rodríguez-Lelis, Sergio Vergara-Limon, Víctor Olivares-Peregrino, Marciano Vargas-Treviño

**Affiliations:** 1 Department of Electronics, National Center for Research and Technological Development, Cuernavaca, Morelos, Mexico; 2 Department of Mechanics, National Center for Research and Technological Development, Cuernavaca, Morelos, Mexico; 3 Autonomous University of Puebla, Puebla, Mexico; 4 Autonomous University Benito Juárez of Oaxaca, Oaxaca, Mexico

## Abstract

**Background:**

Gait analysis and research have been developed to obtain characteristics of movement patterns of people while walking. However, traditional measuring systems present different drawbacks that reduce their use and application. Among those drawbacks one can find: high price, low sampling frequency and limiting number of steps to be analyzed. Traditional measuring gait systems carry out their measurement at frequencies oscillating between 60 to 100 Hz. It can be argued about the need of higher sampling rates for gait measurements. However small displacements of the knee or hip for example, cannot be seen with low frequencies required a more detailed sampling and higher frequency sampling. Bearing this in mind, in this paper is presented a 250 Hz system based on accelerometers for gait measurement, and the particularities of knee and hip angles during gait are highlighted.

**Methods:**

The system was designed with a PCI data acquisition card instrumented with an FPGA to achieve a rate sample of 250 Hz. The accelerometers were placed in thighs and legs to calculate the joint angles of hip and knee in the sagittal plane. The angles were estimated using the acceleration polygon method without integrating the acceleration and without filters.

**Results:**

The gait of thirty healthy people of Mexican phenotype was analyzed over a flat floor free of obstacles. The results showed the gait phases and particularities associated with the walking style and people's laterality; the movement patterns were similar in the thirty persons. Based on the results, the particularities as the maximum amplitude in the angles and the shape in the movement patterns were related to the anthropometry and people phenotype.

**Conclusions:**

The sampling frequency was essential to record 340 samples in single gait cycle and so registering the gait cycle with its particularities. In this work were recorded an average of 8 to 10 gait cycles, and the results showed variation regarding works carried out in biomechanics laboratories; this variation was related to the method and reference frame used to obtain the joint angles and the accuracy of measurement system.

## Background

The process of human locomotion has been the subject of numerous studies in order to comprehend the movements of the lower limb joints during gait. In general, the purpose of the movement analysis during locomotion is to record in real-time the different movements that the joints of the lower limbs perform during gait, without quantifying the forces produced by muscles [1].

In the last two decades technological advances have allowed to develop new biomechanics laboratories which are instrumented with camera systems, force platforms and electromyography systems. These laboratories permit to estimate: the kinematics of the lower extremities in 2D and 3D, ground reaction forces, and muscle activity at each step [2,3]. In these laboratories some patterns have been established to describe the movement of the lower limb joints during the gait cycle [4-8]. These patterns have been considered as standard patterns of normal gait in orthopedics rehabilitation and biomedical engineering because they are representative of people without considering sex, age and anthropometry. The patterns are widely used not only to assess a normal or pathological gait but also to design prosthetics, orthotics and exoskeletons for lower limbs to improve the level in human-machine coordination [9-13]. Biomechanical laboratories have some disadvantages such as: 1) the camera systems are expensive and need complex instrumentation and environment setting, 2) the number of steps that are analyzed are few because the analysis area in the laboratory is limited by the number of cameras and the force plates [2,14-16].

Another method to analyze human motion can be achieved through the portable systems. These systems have been developed as an alternative to gait analysis and reduce some disadvantages of biomechanics laboratories. The portable measuring systems used some inertial sensors like accelerometers, gyroscopes and magnetometers to obtain the joint angles in the lower extremities. The portability of these systems allows to record the kinematics of the lower limbs during several steps. The systems developed with accelerometers measure the relative acceleration in corporal segments of the lower limbs with respect to a framework. The measured acceleration reflected the intensity and frequency in human movements.

The studies made with accelerometers for human movement analysis have increased in the last decade. One aim of these studies is to capture movements of the joints during daily activities to analyze positions and classify the different movements.

The systems instrumented with accelerometers have advantages such as the portability of the system due to the size of the devices and the several gait cycles captured. However, one main disadvantage is to obtain the joint angle through the integrating of angular acceleration or angular velocity [2,9,17].

Willemsen et al. [18] developed a method that estimates the joint angles of the lower limb without integrating the acceleration obtained in accelerometers. This method requires two accelerometer pairs mounted on adjacent segments in the lower limb. Dejnabadi et al. [2] reported a new technique to obtain the joint angles without integrating the acceleration, so the angles are free of any source drift. Kun et al. [19] instrumented accelerometers and magnetometers to estimate the knee kinematics. The method was based on two algorithms: 1) Based on the differences of measurement obtained of fixed sensors, and 2) Based on the difference of measurement of virtual sensors. Both algorithms were used to calculate the angles of flexion-extension, abduction-adduction and inversion-eversion.

Correa and Balbinot [20] developed a measurement system based on accelerometers to analyze the human gait. It is a wireless system with a virtual model of the human body which has a sampling rate of 50Hz. The Accelerometers were placed on the thigh and leg to measure the joint angles in hip and knee during the walking. This system was compared with one videometry based system which showed a variation in the thigh angle of 8.6 degrees, and the leg variation was about 21.8 degrees.

Godwin et al. [21] analyzed the accuracy of the inertial sensors (IMSs). The accuracy was assessed through a planar pendulum in three situations: static, quasi-static and dynamic. The results were compared with a Vicon gold-standard camera system. The errors in quasi-static and static situations were of 0.3 degrees, and in the dynamic test were between 1.9 and 3.5 degrees.

Several studies about motion analysis that have been made with accelerometers have not reported the different phases of the gait cycle yet as the studies made in biomechanics laboratories. The major causes are related with the problems to obtain the joint angles through the acceleration obtained by accelerometers placed at body segments, and by low rate sample achieved by the systems developed.

The main problems of the systems developed with accelerometers are because of the method to obtain the joint angles through the acceleration recorded by the accelerometers, and by the low sampling frequencies achieved for developed systems.

This paper presents the characterization of one gait cycle obtained from a measurement system developed with accelerometers. These inertial sensors have a measurement rate of ±1.7 gravities. The measurement system achieved a sample rate of 250 Hz. The accelerometers were placed on the thighs and legs to get the tilt angle in each corporal segment using the method of polygon of acceleration considering the gravity vector as a reference point. Taking into account that the movements of the joints during walking are cyclical in both lower limbs, the movement patterns in the joints should be similar and then should show alternation in the movements of the right lower limb and the left lower limb. The aim of the system was to record the joint angles during multiple cycles to obtain the motion pattern in the persons, and with it identify the particularities and phases of gait.

## Methods

Human locomotion process is an activity developed by the lower extremities which have the aim to move the center-mass toward the forehead. The movements performed by the joints and corporal segments during gait have a frequency from 5 to 40 Hz [22]. According to [1] some physical activities such as walking, posture and transitional activities can be classified using accelerometers placed in different segments of the body.

### Method to obtain the tilt angle

The measurement electronic systems developed with accelerometers analyze the motion through the acceleration measurement in the body segments of interest. This method measures the acceleration of each segment, along an orthogonal system of reference which reflects intensity and frequency in movements. Thus, to obtain the kinematics of the lower limbs: first the tilt angles of the thighs and legs were obtained independently and subsequently the kinematics to each lower limb was calculated.

**Figure 1:**
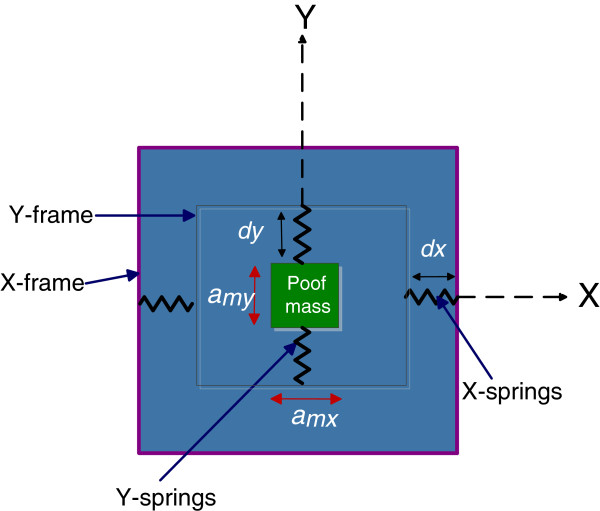
Representation of accelerometer for two-axis.

The tilt angles were obtained with accelerometers of two axes. According to [15] an accelerometer of two axes have one proof mass, two frame and two springs as show Figure 1. The accelerometer measurement the acceleration in the proof mass, which is the sum of specific force and the gravity acceleration by unit mass. If the mass is considered *m* = 1, the acceleration in proof mass is:

(1) $$\overset{\to }{a_{m}}=\overset{\to }{F_{s}}+\overset{\to }{g}$$

Thus, the specific force is given by:

(2) $$\overset{\to }{F_{s}}=k*\overset{\to }{d}+k*\mathrm{dx}=\overset{\to }{a_{m}}-\overset{\to }{g}$$

where *k* is the spring stiffness constant, $\overset{\to }{d}$ is the displacement caused by stretching of the spring, $\overset{\to }{a_{m}}$ is the acceleration of the test- mass and $\overset{\to }{g}$ the gravity.

The acceleration determined by the accelerometer is:

(3) $$\overset{\to }{a}=-g+\overset{\to }{a_{m}}=a_{n}+a_{t}$$

where: *α*_*n*_ is normal acceleration and *α*_*t*_ is tangential acceleration.

In this way, when the acceleration $\overset{\to }{a}$ is the value of gravity, it is considered as constant acceleration and can be determined by:

(4) $$\begin{array}{l} \overset{\to }{a}=-g(\text{sin}\left(\theta \right)+\text{cos}\left(\theta \right)) \end{array}$$

In this case the tilt angle can be estimated through the acceleration polygon method and using the gravity vector like reference point, so the tilt angle is determined by:

(5) $$\theta ={\text{tan}}^{-1}\frac{a_{x}}{a_{y}}$$

where *α*_*x*_ and *α*_*y*_ are the accelerations measured by the accelerometer in the frame x’-y’.

**Figure 2:**
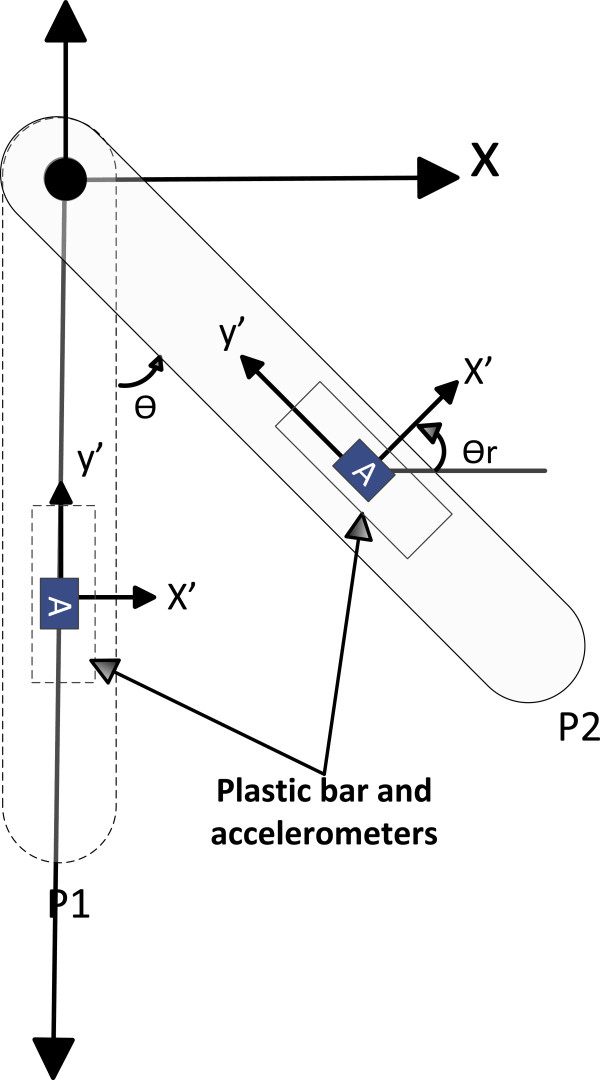
Tilt angle.

Then, to obtain the cinematic of the lower limb, the tilt angles in each body segment were obtained through equation 5. Thus, first the tilt angles of the thighs and legs were obtained independently in each lower limb. The accelerometers were placed in each body segment to obtain its tilt angle as shown in Figure 2. In Figure 2, the bar with reference frame X-Y represents a body segment; the Y-axis of the reference frame is parallel to the gravity vector and the sagittal plane. So, on the bar an accelerometer is placed to obtain the tilt angle. The accelerometer frame is x'-y '. When the bar changes from P1 to P2, the accelerometer´s frame (x´ y y´) suffers rotation respect to the initial frame X-Y. The tilt angle θ can be obtained by equation 5.

$$\theta =\theta_{r}={\text{tan}}^{-1}\frac{a_{x}}{a_{y}}$$

Where *θ* is the tilt angle, *θ*_*r*_ the angle of rotation in the accelerometer´s frame repect to X-Y, *α*_*x*_ the acceleration in *x*-axis and *α*_*y*_ the acceleration in y-axis.

**Figure 3:**
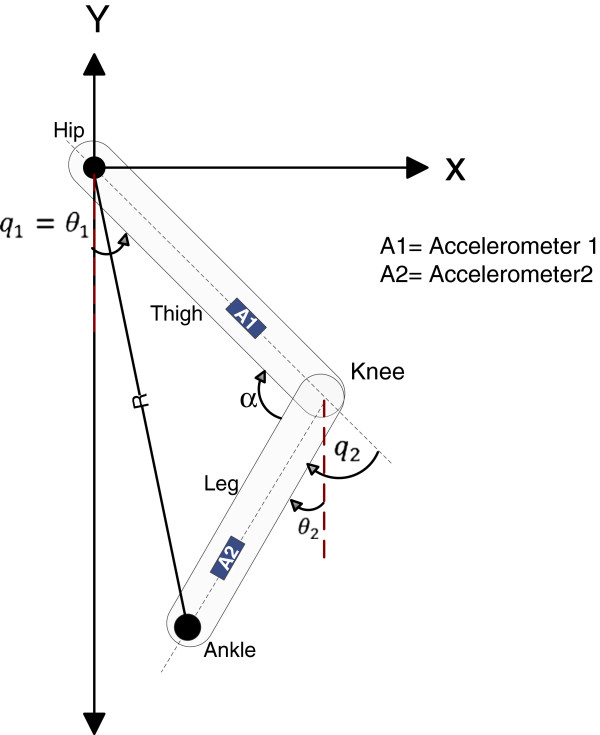
Estimation of angles for hip and knee.

The accelerometer was tied to a flexible plastic bar which was attached to the body segment with elastic tape to avoid the accelerometers change position during gait analysis. In order to obtain the kinematics of the lower limb, it was necessary to set up a new reference frame X”-Y” with origin in the hip joint as shown in Figure 3. The body segments are presented with two rigid links L1 and L2 that represent thigh and leg respectively.

In accordance to [8], the hip angle may be measured in two different ways: 1) the angle between the vertical and femur which is known as absolute angle, and 2) the angle between pelvis and femur known as relative angle. Then considering that during a normal gait, the trunk stays in a perpendicular position; the angle hip was obtained using the first method. In this way to Figure 3 the angle *θ*_1_ is the tilt angle of the thigh and it can be considered like the absolute angle of hip, which is given by:

(6) $$q_{1}=\theta_{1}$$

In similar way to obtain the knee angle, the second method by [8] was used. Firstly the tilt angle of the leg was obtained through the accelerometer 2 (see Figure 3), and finally from L1 projection, the relative angle of the knee can be estimated by:

(7) $$q_{2}=\theta_{1}-\theta_{2}$$

where *q*_2_ is a relative angle of the knee, *θ*_1_ is the tilt angle of the thigh and *θ*_2_ is the tilt angle of the leg.

In Figure 3 the total length of the lower limb is determined by:

(8) $$\alpha ={\text{cos}}^{-1}\left(\frac{{∥L1∥}^{2}+{∥L2∥}^{2}-{∥R∥}^{2}}{2∥L1∥∥L2∥}\right)$$

(9) $$R^{2}=L1^{2}+L2^{2}$$

where L1 and L2 are the length in thigh and leg, and R is the total length.

### Measurement system

**Figure 4:**
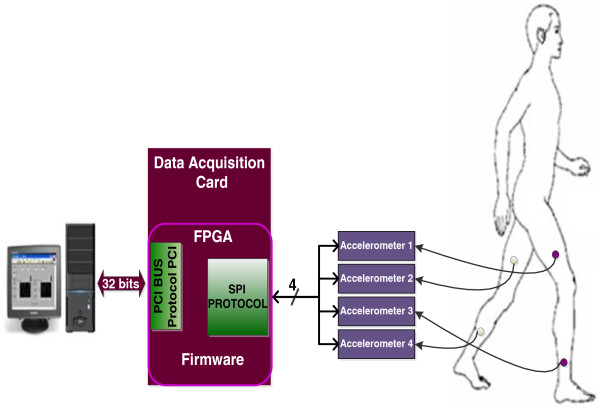
Block diagram of measurement system.

Winter [11] reported that the gait analysis made over 71 Hz provides useful particularities to determine the gait pattern. However, it may be possible that for an upper sample rate, the movement patterns can present some particularities associated to the walking style of people. Therefore, the sampling rate in the measurement system in this work was above 71Hz. Figure 4 presents the block diagram of the measurement system: 1) four accelerometers MEMS as angle sensor; 2) One PCI Card to data acquisition; 3) One computer to visualize and record the data. Some characteristics of electronic devices and system used to develop the measurement system are the following:

SENSORS: four accelerometers ADIS16209 were used to measure the acceleration in the X-Y axes in each body segment; these accelerometers have a measurement range of ±1.7 gravities and 12 bits of resolution (bit LSB is equal to 0.97 mg) and bandwidth of 1.5 KHz. Each accelerometer provides the measured acceleration through the SPI (Serial Peripheral Interface) protocol.

**Figure 5:**
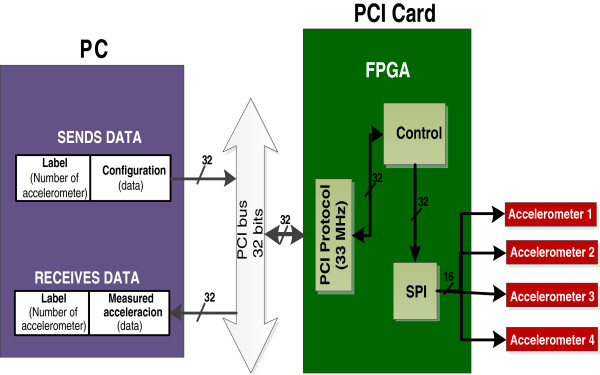
Data transfer between the PC and the PCI card.

**Figure 6:**
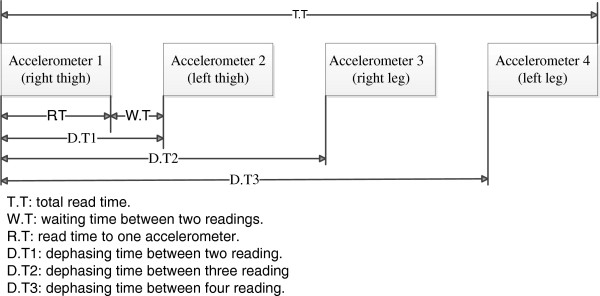
Dephasing between reads.

PCI CARD: the acquisition, processing and control of data flow between the PC and accelerometers were performed with the PCI card through different protocols for data transfer. This PCI card is a modular card of open architecture instrumented with an FPGA of 8256 logics gate which can work up to 100 MHZ. The configuration and reading of each accelerometer, saving and sending the data to a PC for their visualization and also to record them in a file text, was performed by a FPGA via firmware. The firmware was designed in blocks (Figure 5): 1) PCI Block (to read-write): this block was developed for data transfer of 32 bits between the PCI card and the PC through PCI protocol, 2) SPI Block: it was developed to generate the serial handshaking master-slave SPI to communicate the accelerometers and the PCI card, and finally, 3) Control Block: it was developed to control the flow of data of all the blocks. The PCI and SPI blocks were developed through timing diagrams reported by [23,24]. The firmware was designed to configure and to sequentially read the four accelerometers in 831 μs. The sequential reading mode generates a delay between the configuration and the reading time for every accelerometer and one dead time between the readings (see Figure 6). Thus, the maximum delay was 724 microseconds; it was presented between the accelerometer 1 and the accelerometer 4 as shown in Figure 6. Considering that the body movements have a frequency of 4 to 40 Hz, the delay time between readings can not be considered as a mistake to estimate the tilt angle.

**Figure 7:**
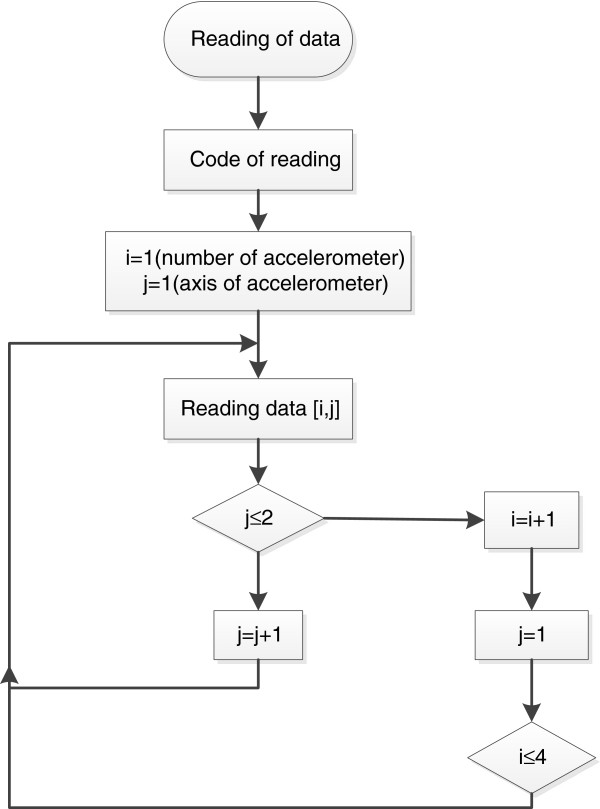
Reading data PC-FPGA.

DATA PROCCESING: one virtual instrumentation software was used to process, to visualize and to watch the obtained data. The software allows data transference by the PCI bus in real-time. The data transference and the sample time between the FPGA-accelerometers and FPGA-PC can be manipulated through software and firmware. Figure 7 presents the flowchart developed to read the data of four accelerometers.

### Assessment of the measurement system

The method to obtain the tilt angle and the measurement electronic system was assessed through elbow of a manipulator robot of three degrees freedom reported by [25,26]. The joint elbow has an incremental encoder with 655,360 p/rev as angle sensor, this is used to position and trajectory control.

The accelerometer was tied on the link of elbow such as shown in Figure 2. The robot elbow was controlled through trajectory tracking control reported by [26]; the base and shoulder robot were fixed at zero degrees without movement. The trajectory was determined by:

(10) $$x=90*\text{sin}\left(t\right)$$

The elbow link described an oscillation from 90 to -90 degrees with the vertically as reference point; the trajectory was constant velocity of 1 rad/sec. The trajectory tracking control presented an error of 0.68 degrees during the evaluation test.

**Figure 8:**
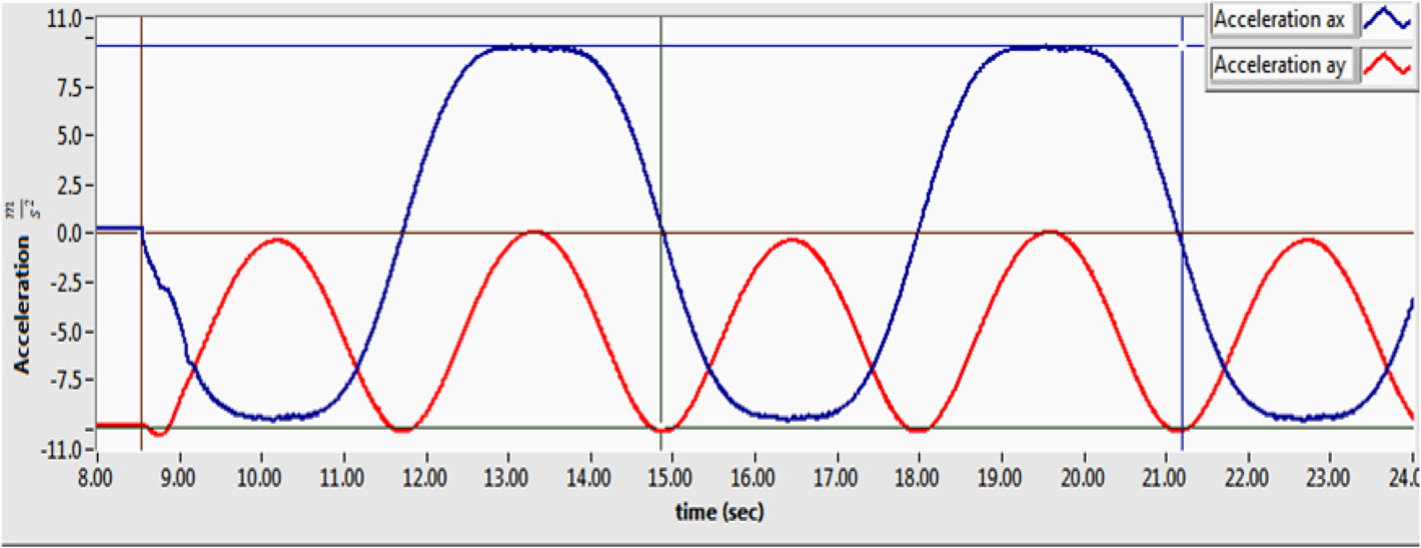
**Accelerations: *α***_***x ***_**blue line and *α***_***y ***_**red line.**

The accelerations *α*_*x*_ and *α*_*y*_ obtained by the accelerometer are shown in Figure 8. The Y-axis presents the acceleration in $\frac{m}{s^{2}}$, and the X-axis present the time in second. In this figure is possible to see that the *α*_*x*_ acceleration (blue line) has a behavior oscillatory that starts at zero, and subsequently, it has an oscillation from 9.8 to -9.8 $\frac{m}{s^{2}}$. Similarly *α*_*y*_ acceleration (red line) has a behavior oscillatory from -9.8 a 0 $\frac{m}{s^{2}}$.

The encoder is used as an angle sensor to measure the position of the elbow, which is used by the control algorithm to estimate the torque. Indirectly, the elbow joint angle was estimated with one accelerometer through equation 5 with the accelerations *α*_*x*_ and *α*_*y*_.

**Figure 9:**
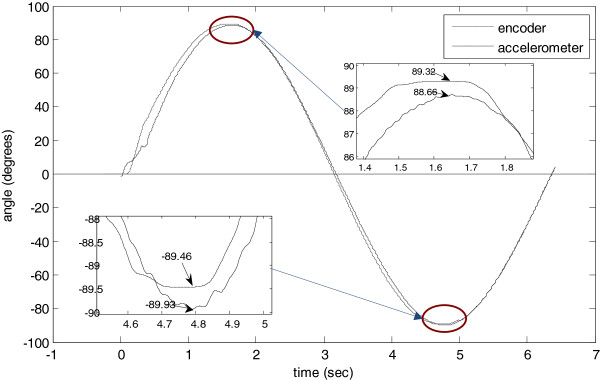
Measuring of the elbow joint angle through encoder and accelerometer.

Figure 9 shows the elbow joint angle obtained with encoder and accelerometer. The angle, obtained with the accelerometer, had one error of 0.75° respect to the encoder.

### Test protocol

The gait analysis was performed on thirty healthy subjects of Mexican phenotype with ages around 20 and 30 years old. Table 1 presents the characteristics people’s sex, age, laterality, height and weight. The length of the thigh and leg were obtained from anthropometric tables considering the body height and weight of every person. Each subject walked an average distance of 10 m in their natural manner. The procedure for analysis was as follows:

• The accelerometers were placed on the thighs and legs as shown in Figure 4.

• One test for adaptation and validation of the system was developed.

• Each subject walked an average distance of 10 m over a flat floor and free of obstacles.

• Two gait test were developed to each subject.

• The subjects stayed without movement for 5 sec at the beginning and end of each gait analysis to establish the reference point.

• The data obtained by the measurement system were recorded in real-time at one text file for subsequent analysis.

**Table 1:** Anatomic and anthropometric data in people analyzed

**Subjects**			**Anatomical data**		
	**Sex (M/F)**	**Age (years)**	**Laterality (right-handed/ left handed)**	**Height (m)**	**Weight (kg)**
Subject 1	M	24	Right-handed	1.90	80
Subject 2	M	25	Right-handed	1.69	90
Subject 3	M	24	Right-handed	1.70	69
Subject 4	M	24	Right-handed	1.70	78
Subject 5	M	28	Left-handed	1.70	79
Subject 6	M	26	Right-handed	1.75	74
Subject 7	F	25	Right-handed	1.65	64
Subject 8	F	26	Right-handed	1.58	52
Subject 9	M	24	Left-handed	1.65	63
Subject 10	F	25	Right-handed	1.60	60
Subject 11	M	24	Right-handed	1.70	71
Subject 12	M	28	Right-handed	1.78	80
Subject 13	F	28	Right-handed	1.56	63
Subject 14	M	25	Right-handed	1.60	65
Subject 15	F	27	Right-handed	1.55	60
Subject 16	M	24	Left-handed	1.60	65
Subject 17	F	25	Right-handed	1.60	75
Subject 18	M	25	Right-handed	1.70	72
Subject 19	M	27	Right-handed	1.62	65
Subject 20	M	27	Right-handed	1.70	80
Subject 21	F	26	Right-handed	1.62	70
Subject 22	F	27	Right-handed	1.55	65
Subject 23	M	25	Left-handed	1.78	95
Subject 24	M	26	Right-handed	1.76	80
Subject 25	M	30	Right-handed	1.76	82
Subject 26	M	27	Right-handed	1.62	85
Subject 27	M	26	Right-handed	1.75	80
Subject 28	M	28	Left-handed	1.80	85
Subject 29	M	23	Right-handed	1.83	84.5
Subject 30	M	28	Right-handed	1.75	80

**Figure 10:**
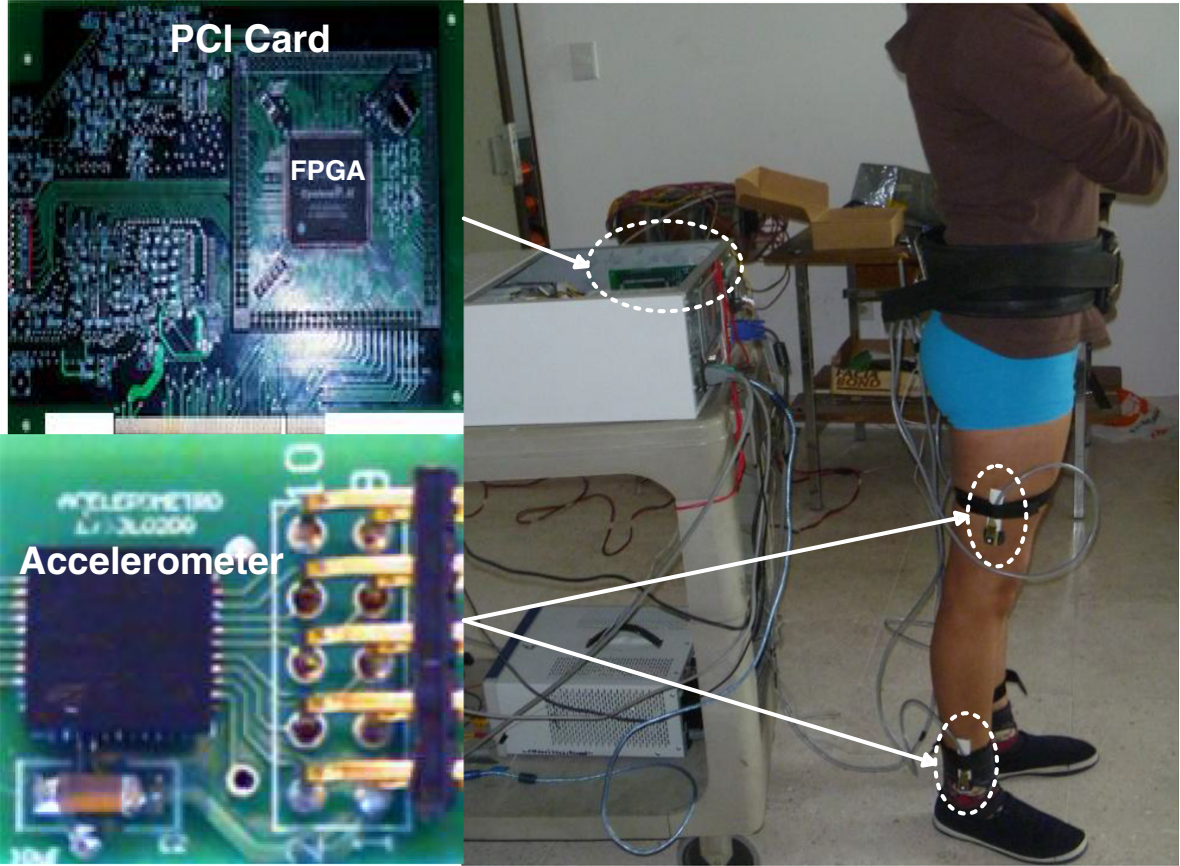
Implementation of the electronic system in a test subject.

Finally, Figure 10 shows the implementation of the electronic measuring system in a test subject and the placement of the accelerometers on the thighs and legs. The portability of the system allowed to record longer distances, and in consequence an increase of the number of steps recorded. The average distance was 20 m, which is around three to four times the distance of regular methods.

The estimation of the tilt angle had a maximum error of 1.5° as a consequence of the alignment of the accelerometers with the reference frame. Thus, the maximum error was 3° and it was presented in the estimation of the knee angle.

## Results

In each test an average of 8 to 10 gait cycles were recorded on both lower limbs. A single gait cycle of the dominant lower limb was selected of each subject. Finally, the gait cycle of a 25 years old healthy woman (subject 7) was used to describe the phases of gait cycle.

**Figure 11:**
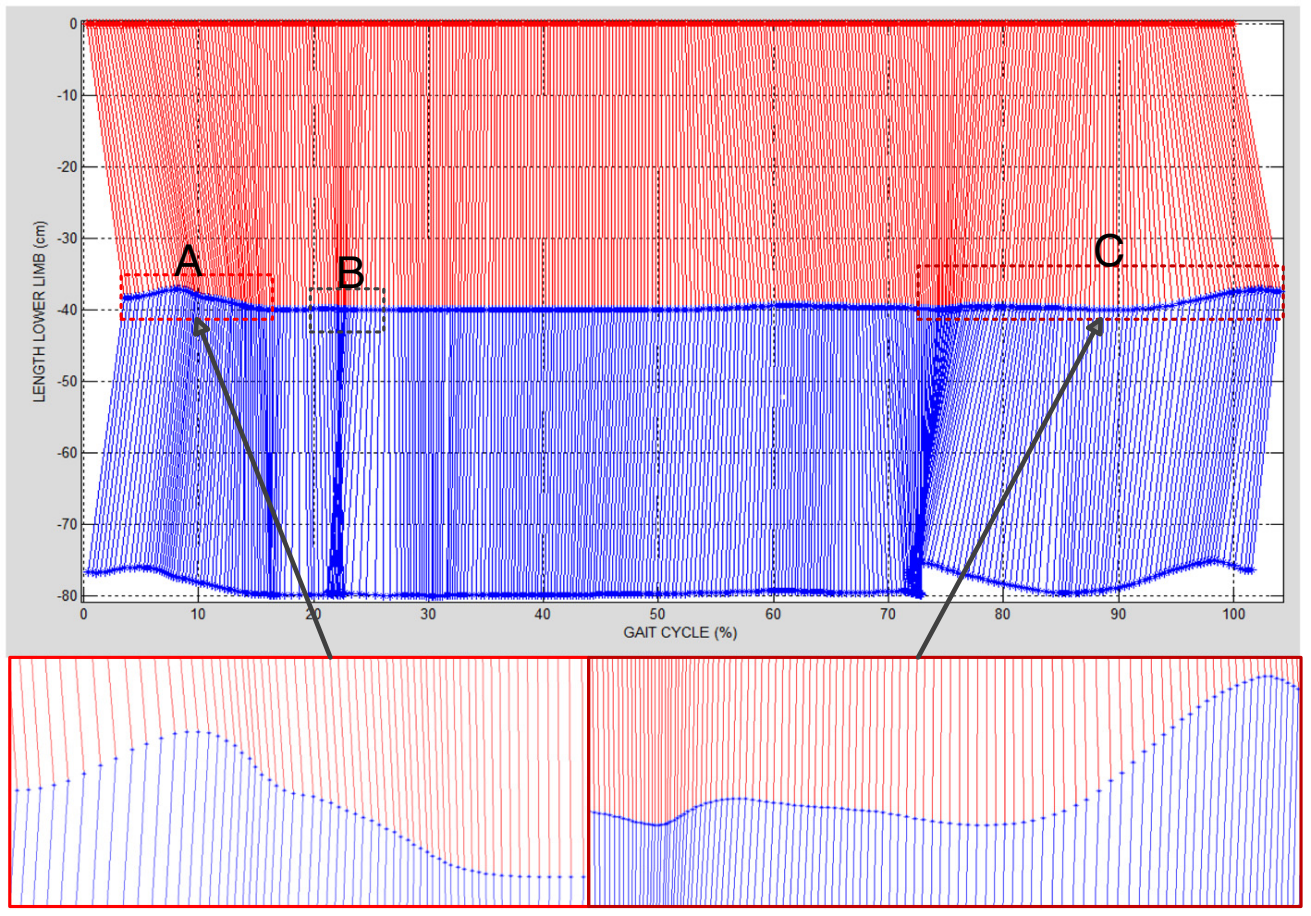
Kinematic of the dominant lower limb (thigh red line and leg blue line) during a gait cycle (subject 7): rectangle "A" movements of flexion-extension at the beginning of gait cycle by loading variation; rectangle "B" motion screw-home of the knee, and rectangle "C" movements of extension-flexion at swing phase.

Figure 11 shows the evolution of the lower limb kinematic during a gait cycle. It allows to watch different positions of thigh (red line) and leg (blue line), and hip and knee angles recorded during gait. Figure 11 reveals three characteristics in kinematics: the first over 10% represented by rectangle A, the second over 20% represented by rectangle B, and the third on 80% represented by rectangle C. The peculiarities at A (10%) and C (80%) are changes in the hip angle sign which is reflected in the knee angle and the lower limb length. According to the results, the particularity B was considered as a possible screw-home motion of the knee and its description is beyond the scope of this work.

The particularity A shows the movements of flexion-extension at the beginning of gait cycle which generates the human body movements toward forehead; this particularity shows the stance phase. The particularity C is associated with the maximum extension of the hip; the particularity C starts at the point where the lower limb thrust, and it concluded until completion the gait cycle; this particularity shows the swing phase. Also, Figure 11 shows that the lower limb stays more time in vertical position (equilibrium movements from 15% to 75%) than in flexion-extension movements.

**Figure 12:**
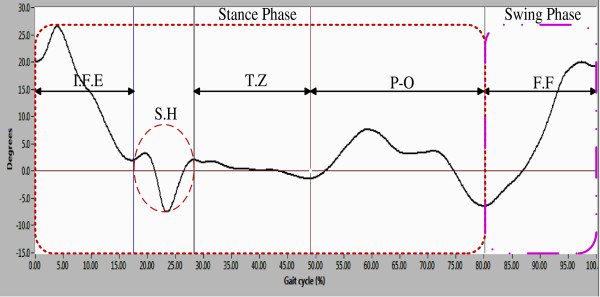
Sagittal plane joint angles of the right hip during a single gait cycle.

Figure 12 presents the joint angles of the right hip (subject 7); it reveals the gait cycle phases: from 0 to 80% is the stance phase, and from 80 to 100% is the swing phase.

To describe the gait cycle, the stance phase was divided into four events: 1) Initial flexion-extension movement (E.I.F): this event begins with a flexion movement as consequence of load variation (loading response), subsequently, one extension movement was made to achieve a vertical position in the lower limb. The amplitude in these movements varies according to the walking style of each person. 2) One possible screw-home movement of the knee (S.H). 3) Transition zone (T.S): In this zone, the foot is in full contact with ground, ready for transition of the complementary lower limb. Finally, 4) Pre_oscilacion (P-O): in this event the lower limb performed movements to be placed in a push position for the next step; the stance phase ends with this event.

Swing phase: this phase presents only one final flexion (F.F); the flexion starts in push position (end of the stance phase) and finishes when the gait cycle ends.

**Figure 13:**
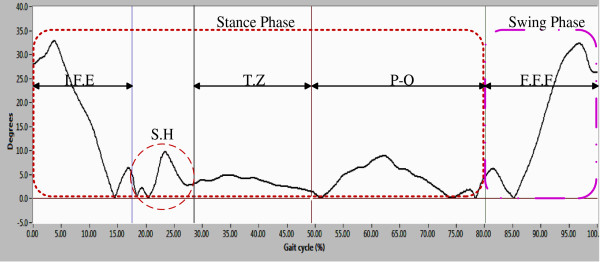
Sagittal plane joint angles of the right knee during a single gait cycle.

Likewise, Figure 13 presents the joint angles of the right knee (subject 7); it shows the gait cycle phases: the stance phase of 0-80%, and swing phase of 80-100% approximately.

To describe the gait cycle, the stance phase was divided into four events: 1) Movement Initial flexion-extension (E.I.F): this event begins with a flexion movement generated in response of variation load (loading response) and subsequently, one extension movement was made to achieve a vertical position of the lower limb. These movements are related with hip movements. 2) One possible screw-home movement of the knee (S.H). 3) Transition zone (T.Z): in this event the lower limb is quasi vertical with angle values less than 10° necessary to keep the body standing. Also during this event was performed the transition of the complementary lower limb. 4) Pre-oscillation stage (P-O): the movements in this stage are to put the lower limb in the push position and with this finish the stance phase.

The swing phase showed only one movement of flexion-extension (F.E.F): first, the knee generates one flexion movement to avoid to trip up and to allow free oscillation subsequently, the knee generates one extension movement which determines the length and end of the step.

**Figure 14:**
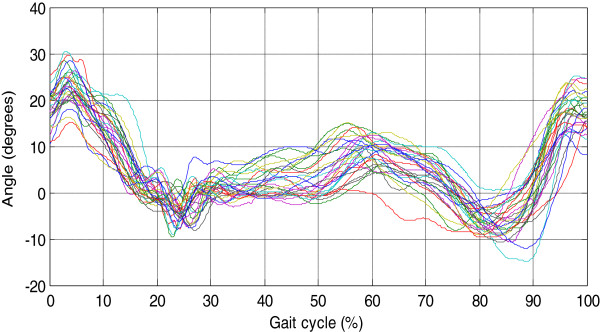
Behavior of hip joint angles of thirty people in the sagittal plane.

The results shown that movement patterns of the dominant lower limb are similar in shape and amplitude. Figure 14 shows the similarities in the patterns of the hip joint angles where an approximate variation of 15° in the maximum amplitudes between gait cycles can be seen.

**Figure 15:**
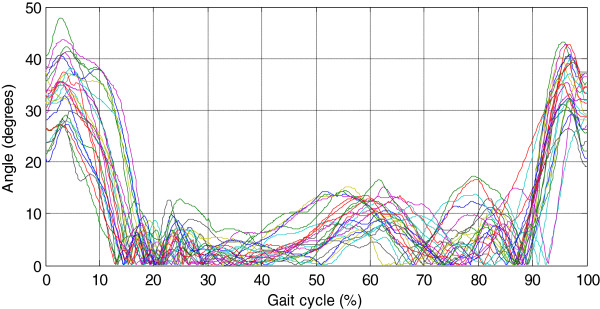
Behavior of knee joint angles of thirty people in the sagittal plane.

Likewise, Figure 15 reveals the similarities in the motion patterns of the knee; the variation is about 20°. In both cases (Figures 14 and 15), the changes were related to walking style, anthropometry, and the phenotype of individuals.

**Figure 16:**
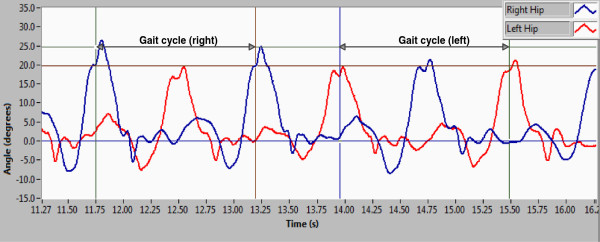
Joint angles of the hip in sagittal plane during one normal gait: blue line right hip and red line left hip.

Generally, the results described rhythmic alternating movements of the lower limbs during walking. The alternation could be observed only in the movements of the hip joints in the same subject. Also, the amplitude of the joint angles of the right hip and left do not have the same amplitude; the dominant limb has a greater amplitude respect to the complementary limb as shown in Figure 16.

Finally the results of the gait analysis are presented in Table 2. This table shows parameters of gait as the laterality of people, the angles of flexion and extension of the hip and knee, also the duration time of the gait cycle. A statistical analysis was developed to obtain the standard deviation in hip and knee angles. In the hip, in stance phase, the standard deviation in extension maximum angle was of 3.004; in the swing phase, the standard deviation in the flexion angle was of 2.569. The knee angles, in stance phase, the standard deviation in extension maximum angle was of 5.425; in the swing phase the standard deviation in the flexion angle was of 4.653.

**Table 2:** Gait test results

**Subjects**	**Laterality**	**Hip**		**Knee**				
	**Variation in maximum amplitude (degrees) and laterality (Right-handed/Left-handed)**	**Maximum flexion (degrees)**	**Maximum extension (degrees)**	**Stance phase**		**Swing phase**		**Gait cycle time (sec)**
				**Flexion (degrees)**	**Extension (degrees)**	**Flexion (degrees)**	**Extension (degrees)**	
Subject 1	2.5, Right-handed	25.5	-11	8	41	37	5	1.412
Subject 2	2, Right-handed	18	-5.5	2	30	30	7	1.444
Subject 3	2, Right-handed	24.6	-10	5	38	32	7	1.508
Subject 4	2, Right-handed	21	-5.5	1	30	30	7	1.516
Subject 5	4, Left-handed	22	-7.3	3	36	40	7	1.412
Subject 6	2, Right-handed	21.8	-9	3	46.5	46.7	5	1.512
Subject 7	3, Right-handed	22.5	-6	3	27	32.4	7	1.396
Subject 8	1.3, Right-handed	21	-9.5	5	35	37	6	1.252
Subject 9	5.2, Left-handed	25.6	-8.5	6	43	43	6	1.236
Subject 10	3.5, Right-handed	24	-5.5	2	34.5	37	5	1.386
Subject 11	2, Right-handed	24.3	-5.3	6	44.5	41.5	5	1.226
Subject 12	2, Right-handed	25.3	-3	5	37.2	40	7	1.576
Subject 13	3, Right-handed	24.2	-4	4	41.7	42	6	1.356
Subject 14	3, Right-handed	52	-6	5	42	40.3	5	1.246
Subject 15	2, Right-handed	23.6	-5	2	32	40.7	9	1.156
Subject 16	2, Left-handed	28.7	-17	8	39	39.8	5	1.286
Subject 17	2, Right-handed	25.5	-12	6	37	40	8	1.296
Subject 18	2, Right-handed	21.2	-4.4	5	34.8	39.3	9	1.316
Subject 19	2, Right-handed	24.4	-5	7	42.4	43	9	1.226
Subject 20	1.5, Right-handed	21.5	-7.5	6	27.3	28.4	6	1.414
Subject 21	2, Right-handed	21.1	-9.1	3	35.5	35.5	3.5	1.284
Subject 22	1.5, Right-handed	18.9	-8.5	6	30.1	30.1	6	1.408
Subject 23	3, Left-handed	28.2	-3.6	7	38.9	39.4	7	1.460
Subject 24	2, Right-handed	19.6	-9.1	7	28.7	31.1	9	1.448
Subject 25	5, Right-handed	21.6	-8.7	7	27.5	29.4	9	1.408
Subject 26	1.7, Right-handed	22.8	-8	7	36	33.2	6.5	1.384
Subject 27	4, Right-handed	24.7	-8.5	3	38.2	34.8	3.7	1.368
Subject 28	2.5, Left-handed	19.8	-6.2	8	32	36.5	12	1.476
Subject 29	1.7, Right-handed	22.9	-10	6	28.5	26.8	8	1.484
Subject 30	2, Right-handed	25.3	-5.3	5	37.2	40	6.5	1.457

## Discussion

In the last decade, accelerometers have been widely used in design of portable measurement systems developed primarily to human motion analysis. Systems have different purpose, these can be utilized to assess physical activity and the inclined of terrains [1,2,27]. However, the accelerometers are the inertial sensors most used to gait analysis. Nevertheless these sensors have some problems to obtain the knee and hip joint angles. The main problem is to obtain the joint angles through acceleration. Some research about gait analysis have reported the joint angles of knee and hip in gait cycle, but these have not described the characterization of events and phases in gait cycle [9,14,19,26]. Djuric-Jovicic et al. [28] reported one method to estimate the joint angles through a system developed with accelerometers which do not need the integration of the acceleration to get the joint angles. The system was evaluated with goniometers and the error was of 6°.

Measurement systems for gait analysis instrumented with cameras are expensive due to the number of cameras and the software utilized for the analysis of movement. The average costs of one system with eight cameras and motion tracking software was published by [29], this system is ideal for human movement analysis. The measurement system developed in this work costs only 7.6% of the camera system reported above. The system achieved a frequency of 250 Hz, and the joint angles were obtained without integrating the acceleration and without using filters.

The angles had an error on account of two factors: 1) the alignment of the accelerometer with the vertical, and 2) the small displacement that could have the accelerometer during analysis, these without considering the error of tilt angle of 0.75°. So the maximum error in the hip angle was 1.55°, and 3.1° (approximately) in the knee angle, the latter was higher because the angle knee depends on the tilt angles in the thigh and leg. The average of the sample record in a single gait cycle was of 340 samples. The aim was obtain the joint angles in knee and hip and its particularity to characterized the even and phases in gait cycle; and additionally see as these could change when the measurement system use a mobile reference frame. The method to calculate the tilt angle only can be considered to angular velocities of 1 rad/sec.

The results described motion patterns of the joint of hip and knee with particularities associated with people walking style. The capture of multiple gait cycles revealed a difference in the maximum amplitudes of the hip joint which was associated with the laterality of the subject. Also, the results indicate that there are not two equal consecutive motion patterns even if it is the same subject.

Regarding the characterization of the gait cycle, several works describe the joint angles of hip and knee in the sagittal plane. These works show a standard movement pattern of the lower limb joints [7,8,11,13,22]. Particularly the work reported by [8] shows the motion pattern of hip and knee in the sagittal plane with the following characteristics: 1) in hip reported three movements: two flexions about 29 degrees, one at beginning and another at the cycle ends; and one extension of -15° about 50% of the gait cycle, 2) in knee reported two flexion-extension movements: one of 0 to 30% of the gait cycle with an amplitude approximately of 15°, and another in 30-100% of the gait cycle with an amplitude of 50°.

In this paper, the characterization of the gait cycle was performed in the sagittal plane. Figures 12 and 13 present hip and knee angles with the gait phases (stance phase and swing phase), as several authors have reported in [7,8,11].

For the characterization of the hip joint angle, it was necessary to divide the gait cycle in 5 events as shown in Figure 12. The gait cycle starts with a flexion movement about 6° which determines the maximum amplitude. This movement is caused by the weight change owing transition of the lower limb. As for the pattern the knee (see Figure 13), it presents two movements of flexion-extension: one of 0-15% and other of 85-100% of the gait cycle. The movements indicate that the gait cycle starts with an angle greater than 15°, and it ends with an angle of similar amplitude. The average angular velocity registered during gait was of 0.7 rad/sec it was presented at stance phase. Based on the obtained results, it was observed that the angles vary as a consequence of the style walking, anthropometric characteristics and people phenotype, but they are similar to the movement patterns.

Finally, the evolution of the joint movement of the dominant lower limb was showed in Figure 11 to reproduce each captured movement by the measuring system. Graphically it was possible to observe that the flexions at the beginning and the end the cycle ends are performed in time short intervals with regard to the movements made in the transition and the movements of equilibrium to keep up body standing.

## Conclusions

In this work the kinematics of the lower limbs was obtained through a measurement system developed with accelerometers. The results described different movement patterns in comparison with the papers reported in literature. These differences are considered as a consequence of the reference frame used to obtain the joint angles. The aim was to obtain the gait cycle with the advantages and disadvantages that the method have.

The results showed the particularities that were related with the laterality of people and one possible screw-home movement of the knee. Moreover, according to the parameter similarities, the characterization could be used to obtain normal gait patterns like the ones obtained in biomechanical laboratories.

The measurement system presented some advantages such as: 1) Portability: it allowed to record several steps during the gait (a average 8 to 10 gait cycles). 2) Cheap system: the electronic devices implemented are low cost and the instrumentation is not complex, furthermore it is not necessary to adapt the system to the environment. 3) The obtaining of the tilt angle without integrating the acceleration: this allowed that the error in the joint angles was related with the accelerometers alignment.

The FPGA instrumented on PCI card allows to increase the number of accelerometers or sensors others without affect the samples per second.

The length of the power cord limited the portability of the system, for this reason in a future work, the accelerometers will be wireless to remove the power cord between the accelerometers and the Pc.

## Competing interests

The authors declare that they have no competing interests.

## Authors’ contributions

FM; Conducted the study, the analysis the data and wrote the manuscript. SV, VO and MV design the measurement system and helped in the interpretation of the results. JR and AC were involved in the design of study, interpreted of results and reviewed the manuscript for scientific content. All the authors read and approved the final manuscript.
